# The Myocyte Expression of Adiponectin Receptors and PPAR*δ* Is Highly Coordinated and Reflects Lipid Metabolism of the Human Donors

**DOI:** 10.1155/2011/692536

**Published:** 2011-01-27

**Authors:** Anna-Maria Ordelheide, Martin Heni, Nadja Gommer, Lisa Gasse, Carina Haas, Alke Guirguis, Fausto Machicao, Hans-Ulrich Häring, Harald Staiger

**Affiliations:** Division of Endocrinology, Diabetology, Angiology, Nephrology, and Clinical Chemistry, Department of Internal Medicine, Eberhard Karls University Tübingen and German Center for Diabetes Research (DZD e.V.), Tübingen, Germany

## Abstract

Muscle lipid oxidation is stimulated by peroxisome proliferator-activated receptor (PPAR) *δ* or adiponectin receptor signalling. We studied human myocyte expression of the PPAR*δ* and adiponectin receptor genes and their relationship to lipid parameters of the donors. The mRNA levels of the three adiponectin receptors, AdipoR1, AdipoR2, and T-cadherin, were highly interrelated (*r* ≥ 0.91). However, they were not associated with GPBAR1, an unrelated membrane receptor. In addition, the adiponectin receptors were positively associated with PPAR*δ* expression (*r* ≥ 0.75). However, they were not associated with PPAR*α*. Using stepwise multiple linear regression analysis, PPAR*δ* was a significant determinant of T-cadherin (*P* = .0002). However, pharmacological PPAR*δ* activation did not increase T-cadherin expression. The myocyte expression levels of AdipoR1 and T-cadherin were inversely associated with the donors' fasting plasma triglycerides (*P* < .03). In conclusion, myocyte expression of PPAR*δ* and the adiponectin receptors are highly coordinated, and this might be of relevance for human lipid metabolism in vivo.

## 1. Introduction

In states of increased circulating nonesterified fatty acids (NEFA), such as fasting, high dietary fat intake, or obesity, appropriate fatty acid oxidation by skeletal muscle and liver is crucial to maintain insulin sensitivity and glucose tolerance. Impairment of fatty acid oxidation, for example, due to genetics, provokes increments in plasma NEFA levels, ectopic lipid deposition in nonfat tissues, enhanced hepatic VLDL production, and finally hyperlipidaemia [1]. Insulin resistance, glucose intolerance, and type 2 diabetes may arise from enhanced fatty acid signalling in skeletal muscle and liver initiated by the elevated plasma NEFA levels per se or by the accumulation of ectopic lipids [2].

Peroxisome proliferator-activated receptor (PPAR) *δ*, a nuclear receptor and transcription factor, represents an important regulator of fatty acid oxidation in rodent and human skeletal muscle. PPAR*δ* is activated by long-chain NEFA and, in concert with coactivator proteins, such as PPAR*γ* coactivator 1, induces the expression of genes involved in cellular fatty acid uptake, *β*-oxidation, and energy uncoupling [3]. Alternatively, muscular fatty acid oxidation is stimulated by adiponectin, an insulin-sensitizing adipokine negatively associated with adipose tissue mass. Adiponectin signalling triggers phosphorylation and inactivation of acetyl-CoA carboxylase promoting a decline in cytosolic malonyl-CoA levels and, thereby, enhancing fatty acyl-CoA import into mitochondria and *β*-oxidation [4, 5]. The functions of adiponectin are mediated via three different receptors, that is, AdipoR1, AdipoR2, and T-cadherin, which are abundant in skeletal muscle. AdipoR1 and AdipoR2 were shown to bind trimeric full-length adiponectin as well as its proteolytically cleaved globular domain, while T-cadherin binds hexameric and high-molecular-weight oligomeric forms of adiponectin [6]. 

Whether the PPAR*δ* and adiponectin receptor signalling pathways are linked at any cellular level is currently unknown. In this gene expression study, we therefore investigated whether the expression of the genes encoding PPAR*δ* (*PPARD*) and the adiponectin receptors AdipoR1 (*ADIPOR1*), AdipoR2 (*ADIPOR2*), and T-cadherin (*CDH13*) is interrelated. To this end, we used in vitro differentiated human skeletal muscle cells (myotubes) from 39 young and healthy human donors. Since basal gene expression of human myotubes was previously shown to reflect in vivo phenotypes of the donors [7–9], we furthermore assessed whether these genes' expression levels associate with in vivo lipid parameters of the donors.

## 2. Materials and Methods

### 2.1. Cell Culture

Primary human skeletal muscle cells were grown from satellite cells obtained from vastus lateralis muscle biopsies and differentiated to myotubes, as previously described [10]. Some myotube cultures were treated for 20 h either with 0.1% DMSO for carrier control or with 1 *μ*mol/L of the PPAR*δ*-specific agonist GW501516 (Alexis Biochemicals, Grünberg, Germany).

### 2.2. Myotube Donors

The 39 donors were young and healthy Caucasian participants of the Tübingen family study for type 2 diabetes and gave informed written consent to the study. All individuals were metabolically characterised by an oral glucose tolerance test, as described earlier [11]. The subject characteristics are given in Table 1. The study was in accordance with the principles of the Declaration of Helsinki, and the protocol was approved by the Ethics Committee of the Medical Faculty of the University of Tübingen.

### 2.3. Quantitative RT-PCR (qPCR)

Myotubes were washed with PBS and harvested by trypsinisation. RNA was isolated with RNeasy columns (Qiagen, Hilden, Germany). Total RNA treated with RNase-free DNase I was transcribed into cDNA using AMV reverse transcriptase and the First Strand cDNA kit from Roche Diagnostics (Mannheim, Germany). qPCR was performed in triplicate with SYBR Green I dye on a LightCycler (Roche Diagnostics, Mannheim, Germany). The primers were purchased from TIB MOLBIOL (Berlin, Germany): *ADIPOR1* mRNA forward 5′-ATTGAGGTACCAGCCAGATG-3′, reverse 5′-GAGGTCTATGACCATGTAGC-3′; *ADIPOR2* mRNA forward 5′-GATTGTCATCTGTGTGCTGG-3′, reverse 5′-CTGGAGACTGGTAGGTATCA-3′; *CDH13* mRNA forward 5′-TGCTGATAACCCTGGAGGAC-3′, reverse 5′-ATGGGCAGGTTGTAGTTTGC-3′; *GPBAR1* mRNA forward 5′-GCTGCTTCTTCCTGAGCCTAC-3′, reverse 5′-GTTGGGAGCCAAGTAGACGAG-3′; *PPARD* mRNA forward 5′-AAGAGGAAGTGGCAGAGGCA-3′, reverse 5′-TGCCACCAGCTTCTTCTTCT-3′; *PPARA* mRNA forward 5′-CCATCGGCGAGGATAGTTCT-3′, reverse 5′-CTGCGGTCGCACTTGTCATA-3′; 28S-rRNA forward 5′-ACGGCGGGAGTAACTATGACT-3′, reverse 5′-CTTGGCTGTGGTTTCGCT-3′. The annealing temperatures were *ADIPOR1* mRNA −66°C; *ADIPOR2* mRNA* −64°C; *CDH13* mRNA −66°C; *GPBAR1* mRNA −68°C; *PPARD* mRNA* −67°C; *PPARA* mRNA* −67°C; 28S-rRNA −63°C. All reactions contained 4 mmol/l MgCl_2_ (reactions marked with asterisk additionally contained 5% DMSO) and were run for 45 cycles. Cellular mRNA contents are given in fg mRNA (or rRNA)/*μ*g total RNA. The basal expression levels are given in Table 1. Furthermore, we performed probe-based qPCR (LightCycler, Roche Diagnostics) using probes from the Universal ProbeLibrary (Roche Diagnostics) and primers from TIB MOLBIOL to determine the mRNA expression levels of *ADIPOR1*, *ADIPOR2*, *CDH13*, *PPARD*, *UCP3*, *TFAM*, *PPARGC1A*, and the housekeeping gene *RPS13* (primer sequences and PCR conditions can be provided upon request).

### 2.4. Oral Glucose Tolerance Test (OGTT) and Hyperinsulinaemic-Euglycaemic Clamp

Both procedures were performed as previously described in detail [11].

### 2.5. Laboratory Measurements

Glucose was determined using a bedside glucose analyzer (Yellow Springs Instruments, Yellow Springs, OH, USA). Insulin was determined by a microparticle enzyme immunoassay (Abbott Laboratories, Tokyo, Japan). NEFA and glycerol were measured using enzymatic assays from WAKO Chemicals (Neuss, Germany) and Sigma (Deisenhofen, Germany), respectively. Triglycerides, total, HDL, and LDL cholesterol were determined with standard colorimetric methods using a Roche/Hitachi analyzer (Roche Diagnostics, Mannheim, Germany). Adiponectin was determined by a radioimmunoassay (Linco Research, St. Charles, MO, USA).

### 2.6. Selection and Genotyping of Single Nucleotide Polymorphisms (SNPs)

To study the influence of genetic variation on *PPARD*, *ADIPOR1*, *ADIPOR2*, and *CDH13* expression, we selected the unlinked *PPARD* SNPs rs2267668 A/G (intron 2) and rs1053049 T/C (3′-untranslated region) and the *ADIPOR1* promoter SNP rs6666089 for which we previously reported in vivo functionality [11–13]. For genotyping, DNA was isolated from whole blood using a commercial DNA isolation kit (NucleoSpin, Macherey & Nagel, Düren, Germany). Genotyping was performed with TaqMan assays (Applied Biosystems, Foster City, CA, USA). All SNPs passed the quality controls. Details on this as well as on minor allele frequencies, genotyping success rates, and Hardy-Weinberg equilibrium are reported in the aforementioned references.

### 2.7. Statistics

To approximate normal distribution, all data were log*_e_* transformed prior to statistical analysis. Two-group comparisons were performed using unpaired Student's *t*-test. To adjust the dependent variable for confounding variables, multiple linear regression models were used (standard least squares method). Stepwise multiple linear regression analysis was performed to identify the best predictor for the dependent variable. A *P* value <.05 was considered statistically significant. The statistical software package JMP 4.0 (SAS Institute, Cary, NC, USA) was used.

## 3. Results

The basal myotube mRNA expression levels were *CDH13* > *ADIPOR1* > *ADIPOR2* > *PPARD* (Table 1). No significant differences were seen in *PPARD*, *ADIPOR1*, *ADIPOR2*, or *CDH13* mRNA levels between myotubes from male versus female donors (Table 1).

**Figure 1:**
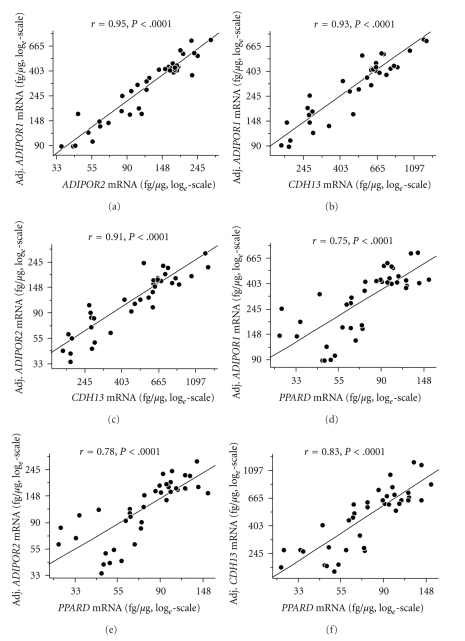
Correlations between *ADIPOR1*, *ADIPOR2*, *CDH13*, and *PPARD* mRNA contents of skeletal muscle cells from 39 human donors (*N* = 39). To approximate normal distribution, all data were log*_e_* transformed prior to statistical analysis. The dependent variable was adjusted for 28S-rRNA using multiple linear regression models.

Using bivariate regression analysis, the mRNA expression levels of all three adiponectin receptors, each normalised for the housekeeping gene 28S-rRNA, were highly interrelated (all *r* ≥ 0.91, all *P* < .0001). Since both the dependent and the independent variable were normalised for 28S-rRNA in these initial analyses, these unusually strong correlations could, theoretically, have reflected the correlation of 28S-rRNA with itself. To avoid this bias, we no longer normalised the mRNA levels for 28S-rRNA but adjusted the mRNA levels of the gene selected as dependent variable for 28S-rRNA using multiple linear regression models. As presented in Figures 1(a)–1(c), the strong correlations between the adiponectin receptors remained unaffected by this procedure. This strengthens the observation that the basal expression of all three adiponectin receptors in human myotubes is highly coordinated. For additional control, we studied the association between the three adiponectin receptors and *GPBAR1* encoding the unrelated membrane-type bile acid receptor known to be expressed in skeletal muscle. After adjustment for 28S-rRNA, none of the adiponectin receptors was significantly correlated with *GPBAR1* (all *P* ≥ .2).

Then, we analysed the association between the adiponectin receptors and *PPARD*. After adjustment for 28S-rRNA, the mRNA levels of all three adiponectin receptors were strongly correlated with *PPARD* expression (Figures 1(d)–1(f)). A similar association between the adiponectin receptors and *PPARA* which encodes PPAR*α*, the second PPAR family member with pro-oxidative properties and of importance in muscle, was not found (all *P* ≥ .2). This underscores the specificity of the association between the adiponectin receptors and *PPARD* at the gene expression level.

**Figure 2:**
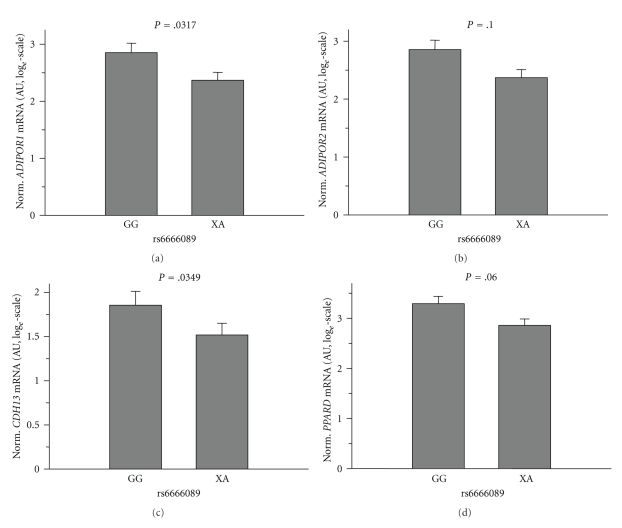
Impact of the *ADIPOR1* promoter SNP rs6666089 on *ADIPOR1*, *ADIPOR2*, *CDH13*, and *PPARD* mRNA contents of human skeletal muscle cells. mRNA data were normalised for 28S-rRNA and are given in arbitrary units (AUs). To approximate normal distribution, mRNA data were log*_e_* transformed prior to statistical analysis. The rs6666089 genotype was determined by TaqMan assay. Since there were only two homozygous carriers of the A-allele among the muscle cell donors, we joined them with the heterozygous subjects to form the “XA” group. Data are presented as means + SE.

**Figure 3:**
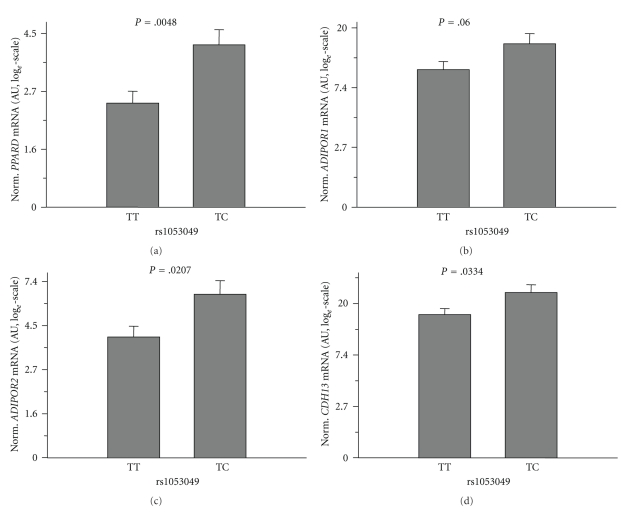
Impact of *PPARD* SNP rs1053049, located in the 3′-untranslated region of the gene, on *PPARD*, *ADIPOR1*, *ADIPOR2*, and *CDH13* mRNA contents of the human skeletal muscle cells. mRNA data were normalised for 28S-rRNA and are given in arbitrary units (AUs). To approximate normal distribution, mRNA data were log*_e_* transformed prior to statistical analysis. The rs1053049 genotype was determined by TaqMan assay. There were no homozygous C-allele carriers among the muscle cell donors. Data are presented as means + SE.

To further strengthen the relationship between the adiponectin receptor and PPAR*δ* genes, we analysed these genes' expression levels according to SNPs in *PPARD* and *ADIPOR1*. As depicted in Figure 2, homo- and heterozygous carriers of the minor allele of *ADIPOR1* SNP rs6666089 (dominant model) revealed significantly reduced expression levels of *ADIPOR1* (*P* = .0317) and *CDH13* (*P* = .0349) and tended to associate with reduced *PPARD* (*P* = .06; *P* for trend in the additive model = .0242) and *ADIPOR2* (*P* = .1; *P* for trend in the additive model = .0284) expression. Heterozygous minor allele carriers of *PPARD* SNP rs1053049 (homozygous carriers of the minor allele were absent in this small group) showed significantly higher expression of *PPARD* (*P* = .0048), *ADIPOR2* (*P* = .0207), and CDH13 (*P* = .0334) and tended to associate with elevated expression of *ADIPOR1* (*P* = .06) as presented in (Figure 3). *PPARD* SNP rs2267668 had markedly weaker effects and, thus, revealed significant association with *PPARD* expression only (*P* = .0258) (data not shown). These results support the idea that the expression of the adiponectin receptor and PPAR*δ* genes is highly coordinated. 

Using stepwise multiple linear regression analysis, we observed that *PPARD* is, independently of the other genes, a significant determinant of *CDH13* (*P* = .0002), but not of *ADIPOR1* or *ADIPOR2* (both *P* > .05). This suggested that *CDH13* represents a novel PPAR*δ* target gene. To address this issue, myotubes were treated for 20 h with the PPAR*δ*-specific agonist GW501516 (1 *μ*mol/l) and DMSO (0.1%) for carrier control. However, GW501516 treatment did not significantly increase *CDH13* mRNA expression as compared to DMSO (*P* = .8, *n* = 16), whereas it increased the expression of the described PPAR*δ* target genes *PDK4* and *ANGPTL4* (for data, see [9]).

To assess whether the highly coordinated expression of *PPARD* and the adiponectin receptor genes—all located on different chromosomes—is caused by a common regulatory miRNA, we screened the miRBase Targets database, Release Version v5 (at http://microrna.sanger.ac.uk/targets/v5/), for common miRNA binding sites in the four genes. In *ADIPOR2*, only one putative binding site was identified, namely, for miRNA-617. The other genes, however, did not reveal any bona fide binding site for miRNA-617. Thus, miRNAs are improbable to represent the unifying link between these genes. To approach whether the genes are under the control of a common transcription factor, we performed in silico analyses of the sequences spanning from 3 kb upstream to 1 kb downstream of the transcription initiation sites. Using AliBaba 2.1 (at http://www.gene-regulation.com/pub/programs/alibaba2/ index.html?), PROMO 3.0 (at http://alggen.lsi.upc.es/ cgi-bin/promo_v3/promo/promoinit.cgi?dirDB=TF_8.3/), MATCH 1.0 (at http://www.gene-regulation.com/cgi-bin/ pub/programs/match/bin/match.cgi/), and P-MATCH 1.0 (at http://www.gene-regulation.com/cgi-bin/pub/programs/ pmatch/bin/p-match.cgi/) freeware for prediction of transcription factor binding sites, we identified three transcription factors, that is, signal transducer and activator of transcription 5A, specificity protein 1, and CCAAT/enhancer-binding protein *α*, with putative binding sites in all four gene sequences. RNA interference-mediated knockdown of these candidates in C2C12 myocytes did however not affect the expression of *PPARD* or the adiponectin receptor genes (data not shown). Additionally, we knocked down FoxO1, one of the known transcriptional regulators of the murine *Adipor1* and *Adipor2* genes [14]. This manipulation did however not affect the expression of the adiponectin receptor and PPAR*δ* genes either (data not shown). Thus, the common upstream regulatory mechanism remains elusive.

Since both adiponectin and PPAR*δ* signalling regulate muscle metabolism and mitochondrial biogenesis, we investigated whether the expression of genes crucially involved in these processes, that is, *PPARGC1A* (encoding PPAR*γ* coactivator 1*α*), *UCP3* (encoding uncoupling protein 3), and *TFAM* (encoding mitochondrial transcription factor A), is correlated with the expression of *ADIPOR1*, *ADIPOR2*, *CDH13*, and *PPARD*. After adjustment for the housekeeping gene, we found significant associations of *ADIPOR1*, *CDH13*, and *PPARD* expression levels with *PPARGC1A*, *UCP3*, and *TFAM* (all *P* < .045). *ADIPOR2* was significantly associated with *TFAM* only (*P* = .007). This strengthens the role of these receptors in mitochondrial oxidative metabolism.

**Figure 4:**
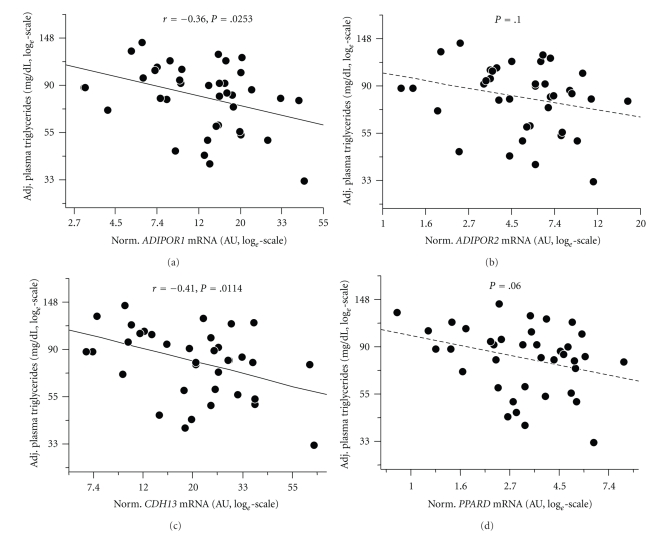
Association of myotube *ADIPOR1*, *ADIPOR2*, *CDH13*, and *PPARD* mRNA contents with fasting plasma triglyceride concentrations of the 39 human donors (*N* = 39). mRNA data were normalised for 28S-rRNA and are given in arbitrary units (AUs). To approximate normal distribution, all data were log*_e_* transformed prior to statistical analysis. Plasma triglycerides were adjusted for gender, age, and BMI using multiple linear regression models.

Finally, we assessed whether the basal myotube expression levels of *PPARD* and the adiponectin receptor genes are of relevance in vivo and reflect fasting plasma lipid parameters of the myotube donors. After adjustment for gender, age, and BMI, the fasting plasma triglyceride concentrations were inversely associated with the *ADIPOR1* and *CDH13* mRNA contents normalised for 28S-rRNA (Figures 4(a) and 4(c)). Moreover, even though not significant, the *ADIPOR2* and *PPARD* mRNA contents tended to inversely associate with plasma triglycerides (Figures 4(b) and 4(d)). No significant associations were detected with fasting plasma NEFA, glycerol, total-, HDL-, or LDL-cholesterol concentrations (all *P* > .1).

In addition, we assessed these genes' association with in vivo parameters of glucose metabolism. However, we did not detect any significant association of *PPARD* and adiponectin receptors expression with fasting insulin concentrations (all *P* ≥ .3), fasting and 120-min glucose concentrations (all *P* ≥ .3), or with insulin sensitivity indices derived from the OGTT and the hyperinsulinaemic-euglycaemic clamp (all *P* > .5), respectively. Serum adiponectin levels did not associate with the expression of *PPARD*, *ADIPOR1*, *ADIPOR2*, or *CDH13* either (all *P* ≥ .3).

## 4. Discussion

In this study, we could demonstrate, also by assessing SNP effects, that two signalling pathways known to mediate lipid-burning and formerly considered independent, that is, the PPAR*δ* and the adiponectin pathway, are closely linked at the gene expression level. The most obvious mechanism possibly underlying this phenomenon, that is, transcriptional regulation of the adiponectin receptor genes by PPAR*δ*, could be excluded since PPAR*δ* activation using a specific and well-described pharmacological agonist had no effect on adiponectin receptor expression. Therefore, we assessed other conceivable upstream regulatory factors. Using RNA interference, transcription factors identified as putative candidates for binding to all four promoters, such as signal transducer and activator of transcription 5A, specificity protein 1, and CCAAT/enhancer-binding protein *α*, turned out not to be involved in this highly coordinated gene regulation, neither did FoxO1, a known transcription factor regulating the murine *Adipor1* and *Adipor2* genes. miRNAs targeting all four genes could also not be identified. Thus, the unifying regulatory mechanism responsible for our observation remains to be uncovered in further studies addressing other, for example, epigenetic mechanisms (DNA methylation, histone modification, and nucleosome positioning).

Most importantly, we could demonstrate that myotube expression of *PPARD* and the adiponectin receptors inversely reflects the fasting plasma triglyceride concentration of the donors. There may be two explanations to this: either circulating triglycerides via their products of lipolytic breakdown, that is, via NEFA, affect the expression of *PPARD* and the adiponectin receptor genes or increased expression of these genes reduces the plasma triglyceride concentration. At least for *PPARD*, we were recently able to show that, with the only exception of stearate, no long-chain fatty acid is able to change its expression [9]. Thus, an influence of triglyceride-derived NEFA on these genes' coordinated expression appears less plausible. Instead, it is well known that the adiponectin and PPAR*δ* pathways stimulate *β*-oxidation and, in this way, may reduce the plasma triglyceride concentration of the donors. Since plasma triglycerides are a well-known readout of the hepatic lipid load [15], this association is in line with the observation that an impairment of fatty acid oxidation provokes increments in plasma NEFA levels, ectopic lipid deposition in nonfat tissues, and enhanced hepatic VLDL production [1] and underscores the idea of a muscle-liver axis with muscle fatty acid oxidation being an important determinant of hepatic lipid storage. In this context, metabolic signals from *β*-oxidation (acyl-carnitines) or muscle-derived humoral mediators (myokines) may represent interesting candidate mediators of muscle-liver crosstalk. Finally, this translational finding provides evidence that our in vitro data are of relevance for humans in vivo and may explain the biological variance between individuals in terms of good versus bad fat burners.

## 5. Conclusions

Expression of *PPARD*, *ADIPOR1*, *ADIPOR2*, and *CDH13* in human skeletal muscle cells is highly coordinated, and this might be of relevance for human lipid metabolism in vivo, as reflected by these genes' consistent inverse association with plasma triglycerides. Thus, the upstream regulatory factor(s) responsible for this coordinated gene expression could represent promising future targets for the control of circulating lipids and hepatic fat load.

##  Conflict of Interests

The authors have no conflict of interests related to this paper to declare.

## Figures and Tables

**Table 1:** Clinical characteristics and myotube gene expression data of the donors (*N* = 39).

Parameter	Women	Men	*P*_1_	*P*_2_
*N*	18	21	—	—
Age (y)	28 ± 7	25 ± 4	.12	—
BMI (kg/m²)	24.1 ± 4.0	22.8 ± 2.9	.2	.5
Body fat (%)	27 ± 8	18 ± 6	.0002	.0006
Waist-hip ratio	0.79 ± 0.09	0.84 ± 0.05	.0155	.0006
Fasting plasma glucose (mM)	4.75 ± 0.40	4.76 ± 0.47	1.0	.8
120-min plasma glucose (mM)	5.24 ± 1.25	5.40 ± 1.15	.6	.5
*ADIPOR1* mRNA	369 ± 198	343 ± 185	1.0	—
*ADIPOR2* mRNA	149 ± 75	131 ± 64	.7	—
*CDH13* mRNA	601 ± 324	537 ± 295	.8	—
*GPBAR1* mRNA	3.16 ± 0.94	2.68 ± 0.70	.16	—
*PPARD* mRNA	81.8 ± 40.2	84.6 ± 38.4	.8	—
*PPARA* mRNA	47.8 ± 11.0	49.0 ± 8.5	.6	—
28S-rRNA	2.70 ± 1.19 × 10^6^	2.61 ± 1.18 × 10^6^	.9	—

Data are given as means ± SD. Gene expression data are given in fg mRNA (or rRNA)/*μ*g total RNA. Statistical analysis was performed after ln-transformation of the data. *P*_1_: unadjusted *P* values (Student's *t*-test); *P*_ 2_: *P* values after adjustment (multiple linear regression analysis): BMI, body fat, and waist-hip ratio were adjusted for age; plasma glucose concentrations were adjusted for age and body fat.

## Abbreviations

AdipoR: Adiponectin receptor
NEFA: Nonesterified fatty acids
PPAR: Peroxisome proliferator-activated receptor.
